# The Relationship Between Value Orientations and Personal Readiness for Activity in Youth From Russia, Kazakhstan and Latvia

**DOI:** 10.11621/pir2021.0108

**Published:** 2021-06-30

**Authors:** Ekaterina I. Perikova, Inna V. Atamanova, Sergey A. Bogomaz, Baizhol I. Karipbayev, Tatyana S. Filippova, Diana Zagulova

**Affiliations:** a Saint Petersburg State University, Saint Petersburg, Russia; b Tomsk State University, Tomsk, Russia; c Academician E.A. Buketov Karaganda University, Karaganda, Kazakhstan; d Karaganda Technical University, Karaganda, Kazakhstan; e Baltic International Academy, Riga, Latvia

**Keywords:** value orientations, cultural dimensions, psychological system of activity, post-Soviet countries, personal readiness for activity

## Abstract

**Background:**

The development of high-quality human capital is an important objective that involves value orientations, cultural dimensions and psychological characteristics of activity. This article presents a cross-cultural comparison of value orientations and psychological parameters of activity among youth from Russia, Kazakhstan, and Latvia.

**Objective:**

The study addressed three questions: (1) Are there values and attitudes related to the readiness for activity among youth in the three countries? (2) Are there any differences between values and parameters of the psychological system of activity in the Russian, Kazakhstani and Latvian samples? (3) What values and attitudes predict the youth’s readiness for activity in each country?

**Design:**

University students from Russia, Kazakhstan and Latvia were invited to participate in the study. The study sample was selected according to age, sex and period of living in the country. Value orientations, cultural dimensions and attitudes were measured by the Values Survey Module, World Values Survey questionnaire, The Subjective Evaluation of Basic Values Realisability. Personality Research Form, Quality of Life Enjoyment and Satisfaction Questionnaire, Subjective Happiness Scale, Self-Organisation of Activity Questionnaire, Differential Test of Reflexivity, and Satisfaction with Life Scale questionnaires were applied to evaluate the psychological parameters of activity. To analyse the relationship between value orientations and psychological parameters of activity, we used analysis of variance, Pearson’s correlation coefficient and stepwise linear regression.

**Results:**

The cross-cultural variance was established for most values and cultural dimensions in the Russian, Kazakhstani, and Latvian samples, but Personal readiness for activity only differed on the tendency level between the Kazakhstani and Latvian samples. Different values and attitudes accounted for near 57% of the Personal readiness for activity index in Russia and Latvia, but just less than 29% in Kazakhstan.

**Conclusion:**

The activity of university students from Russia depends on their need for achievement and level of happiness. In the Kazakhstani and Latvian samples, the most important factor was the quality of life enjoyment and satisfaction index.

## Introduction

Traditionally, the definition of social progress and human capital as its part has been based on economic terms (a so-called GDP-oriented approach). However, a more people-oriented approach has emerged amongst scholars over the last decades. There are a growing number of published studies that focus on high-quality human capital. These aim at exploring social progress index (Porter, 2013), social capital (Kwon, Heflin & Ruef, 2013; Leyden & Link, 2015), personal potential (Leontiev, 2011), professional self-determination (Alimbayeva, Baimukanova, Sabirova, Karipbaev & Tamabayeva, 2018) and others. Thus, addressing the issue of youth’s value orientations, cultural dimensions and psychological characteristics of activity in the context of their personal and professional development seems to be relevant to the modern world’s challenges.

Cultural dimensions are supposed to contribute to our better understanding of the interplay between personal and environmental aspects when analysing the problem of high-quality human resource development (Atamanova, Bogomaz & Filippova, 2019). Evidence from the various literature is that one’s beliefs and evaluations about diversity do affect one’s perceptions and behaviour (Bell, Connerley & Cocchiara, 2009; Mor Barak, 2014). Also, a positive approach to diversity leads to better outcomes (Homan, van Knippenberg, Van Kleef & De Dreu, 2007, Lauring & Selmer, 2013). Various measurement models of national culture have been created over the past decades; the development of theoretical models went from identifying a list of parameters by which one can compare different cultures to identifying factors of a higher level. These factors represent cultural values, “the principles and fundamental convictions which act as general guides to behaviour, the standards by which particular actions are judged to be good or desirable” (Halstead & Taylor, 2000, p. 169). In other words, each value guides our behaviour and could regulate our activity and readiness for it. The most dominant models of cultural dimensions, which gained recognition and popularity, are the following: Hofstede’s (1983), Schwartz’ (1994), and Inglehart’s (1997) models.

The studies conducted highlighted the role of young people’s basic values and their subjective evaluation of possibilities to realise these values in their sociocultural environments (Bogomaz, Kozlova & Atamanova, 2015). Bogomaz et al. (2015) also showed that the higher a young person’s personal potential, the more possibilities they revealed in their local settings for their personal and professional development. Baumann and Winzar (2017) point out that the extent to which values drive behaviour is a function of the circumstances in which individuals find themselves and the relative importance of competing values in particular circumstances. Decades of research have shown that the relationship between attitudes and behaviour is complicated (Ajzen, 1988; Glasman & Albarracín, 2006), so an intermediary linking value and behaviour could be a psychological parameter characterising one’s activity. Such a parameter for integrating one’s value orientations, cultural dimensions and psychological characteristics of activity could be one’s personal readiness for activity (Atamanova et al., 2019). Our studies show that the index of personal readiness for activity depends on the innovativeness index, traditional values, openness to experience, index of motivation, and maintaining: the individual’s adherence to the values of survival negatively affects the manifestation of personal readiness for activity (Buravleva & Bogomaz, 2020). Thus, personal readiness for activity seems to be a universal characteristic and it could accompany both innovative and traditional activity. Also, personal readiness for activity correlates with emotional intelligence (Bogomaz, Boyko & Yashina, 2019).

Accordingly, it is captivating to explore cultural dimensions of countries that were recently a single unit. The collapse of the Soviet Union in 1991 generated a unique situation when 15 countries simultaneously started their pathways to building independent national communities (Atamanova et al., 2019). The 30 years of post-Soviet development in these countries were full of political, cultural, and economic transformations that brought many changes to the way of life and the population’s mentality. Since all the former Soviet Republics removed themselves from the USSR, they may have been strongly influenced by the influx of western culture. The effect of major political changes on the higher education of post-Soviet states and youth’s values were explored in studies by Yakavets (2016), Azimbayeva (2017), Fedotova (2017), and Mykhailenko, Blayone, Usca, Kvasovskyi & Desyatnyuk (2020). However, cultural and psychological differences are not particularly well researched because these changes are very slow due to the significance of local cultural values. However, we believe that enough time has passed since the collapse of the Soviet Union to see cultural and psychological differences among young people living in various post-Soviet countries.

Today’s young generation in the former Soviet Union countries is an indicator of the social and economic alterations that have occurred since the turbulent turn of the century. Youth is known to be the age category most sensitive to change, so it represents a major research interest in the context of the future trends of social development. A deeper understanding of the cultural context requires a research focus on what is going on in the countries. However, youth’s personal and professional development intertwines with each country’s high-quality human capital from a long-term perspective.

Our research includes youth from three ex-Soviet Union countries with different economic and political pathways to building independent national communities: Russia, Kazakhstan, and Latvia. Also noticeable is the geographical differences of these countries: Kazakhstan is in Asia, Latvia is in Europe, and Russia is in both Asia and Europe.

Our objectives were to test hypotheses of cross-cultural differences in value orientations and readiness for activity among the Russian, Latvian, and Kazakhstani samples made up of university students. This would allow a differentiated approach to discovering the relationship between these individual personality characteristics and students’ personal and professional development activities. The study addressed three questions: (1) Are there values and attitudes related to the readiness for activity among youth in the three countries? (2) Are there any differences between values and parameters of the psychological system of activity in the Russian, Kazakhstani and Latvian samples? (3) What values and attitudes predict youth’s readiness for activity in each of the countries?

## Methods

We used several existing or specially developed scales to analyse value orientations and attitudes in these three countries and (2) psychological parameters of activity. Value orientations and attitudes included indicators such as traditional values versus secular-rational values (T/S-RV), the need for achievement and affiliation, subjective evaluation of possibilities for basic values realisability in the local settings, quality of life enjoyment and satisfaction and subjective happiness, and cultural values, namely Hofstede’s six dimensions. Psychological parameters of activity include purposefulness, planning, systemic reflection and satisfaction with life, and the personal readiness for activity index, which is the average of the four parameters mentioned. In order to estimate all these psychological characteristics, the study participants were asked to fill out several questionnaires. The internal consistency coefficients (Cronbach’s alpha) for each dimension are presented for all the subsamples to be analysed and the total sample, respectively, in Table 1. Cronbach’s alphas ranged from .91 to .65; such scores are considered to be acceptable values (Taber, 2018).

**Table 1:** Cronbach’s alpha coefficients for study variables

Study variables	Russia	Kazakhstan	Latvia	Total sample
T/S-RV	.67	.65	.67	.68
Need for achievement	.68	.65	.63	.66
Need for affiliation	.76	.67	.83	.75
SEBVR index	.88	.87	.88	.88
MPrS	.77	.65	.68	.73
MPuS	.66	.65	.67	.66
MVS	.70	.73	.84	.73
MES	.80	.75	.72	.78
Q-LES-Q index	.88	.91	.88	.89
Physical health	.82	.72	.81	.79
Subjective feelings	.84	.84	.84	.82
Leisure time activities	.75	.68	.68	.67
Social relationships	.76	.76	.69	.68
Subjective Happiness	.85	.82	.81	.84
Purposefulness	.83	.82	.72	.79
Planning	.88	.82	.85	.86
Systemic reflection	.78	.79	.69	.77
Satisfaction with life	.77	.83	.84	.81
Personal readiness for activity index	.84	.88	.78	.86

*Note. T/S-RV = Traditional values versus Secular-rational values. SEBVR = Subjective Evaluation of Basic Values Realisability. MPrS = Metavalue of professional self-realization. MPuS = Metavalue of public self-realization. MVS = Metavalue of vital self-realization. MES = Metavalue of existential self-realization. Q-LES-Q = Quality of Life Enjoyment and Satisfaction Questionnaire*.

1. Values Survey Module (VSM 2013) by Hofstede (Hofstede & Minkov, 2013). This consists of 24 items with a 5-point Likert scale response format, ranging from “*of utmost importance*” to “*of very little or no importance*”. VSM 2013 Hofstede’s six dimensions of culture values entailed the independent variable values. The dependent variables included the Power distance index (PDI), Individualism index (IDV), Masculinity index (MAS), Uncertainty avoidance index (UAI), Long-term orientation index (LTO), and Indulgence versus restraint index (IVR). A reliability test for the VSM 2013 should be based on the country mean scores; however, the number of countries is insufficient in our case. According to Hofstede and Minkov (2013), the reliability of the VSM at the country level is taken for granted, and can indirectly be shown through the validity of the scores. To estimate this, we performed correlation analysis between value orientations, attitudes and psychological parameters of activity, which showed a satisfactory level of consistency (Table 3).
2. World Values Survey questionnaire (WVS) by Inglehart (Inglehart & Welzel, 2005), modified by Khabibulin (2015). The scale consists of 13 items with a 7-point Likert scale response format, ranging from “*totally disagree*” to “*absolutely agree*”. The scale includes two axes: Traditional values versus Secular-rational values (T/S-RV) and Survival values versus Self-expression values (S/S-EV). In our study, we only used the T/S-RV dimension since the Cronbach’s alpha values for the S/S-EV scale were inadequate for one of the subsamples.
3. The Subjective Evaluation of Basic Values Realisability (SEBVR) technique developed by Bogomaz (for more details, see Bogomaz. 2014; Atamanova et al., 2019; Filenko, Atamanova, Bogomaz, 2020) for examining one’s subjective evaluation of possibilities for basic values realisability in the local settings. The Subjective Evaluation of Basic Values Realisability technique is a 20-item instrument based on the semantic differential method originated by Osgood and analogous to the Noetic Orientations Test. The task is to evaluate each bipolar set using a 7-point Likert-like scale (3-2-1-0-1-2-3). For this study, four Metavalues of professional self-realisation (MPrS), Metavalue of public self-realisation (MPuS), Metavalue of vital self-realisation (MVS) and Metavalue of existential self-realisation (MES)) and the SEBVR index were used (see Filenko, Atamanova, Bogomaz, 2020).
4. The Personality Research Form (PRF) developed by Jackson (1984), in Kondakov’s modification (1998), is a 6-item questionnaire using a 5-point response scale, ranging from “*strongly disagree*” to “s*trongly agree*”. This version of the PRF estimates two subscales: Need for achievement and Need for affiliation.
5. A short version of the Quality of Life Enjoyment and Satisfaction Questionnaire (Q-LES-Q) is a self-report instrument by Ritsner and colleagues (2005) in Rasskazova’s adaptation (2012). The questionnaire consists of 17 items with a 5-point response scale from “*hardly ever*” to “*constantly*”. The Q-LES-Q includes five measures: Physical health, Subjective feelings, Leisure time activities, Social relationships, and the Q-LES-Q index.
6. The Subjective Happiness Scale (SHS) was constructed by Lyubomirsky and Lepper (1999) and adapted by Leontiev and Osin (2000). It was designed to assess a person’s current psychological state. The questionnaire is a 4-item measure using a 5-point response scale from “*totally disagree*” to “*absolutely agree*”. However, we used only three items according to the recommendation from Leontiev (Osin & Leontiev, 2020).
7. The Self-Organisation of Activity Questionnaire was developed by Mandrikova (2010) to evaluate the maturity of tactical planning and strategic goal-setting skills. The questionnaire was extended from the basis of the Time Structure Questionnaire – TSQ. The Self-Organization of Activity Questionnaire consists of 25 items. For our study, we used only two subscales: Purposefulness (one’s ability to concentrate on a goal) and Planning (one’s involvement in tactical planning according to certain principles). The items were evaluated by a 5-point Likert-like scale ranging from “*completely disagree*” to “*completely agree*”.
8. The Differential Test of Reflexivity (DTR) by Leontiev and Osin (2014) is a 30-item questionnaire using a 4-point response scale, operationalising Leontiev’s 3-component model of reflexive processes. According to the model, systemic reflection (a tendency to look at oneself within the context of situations and life in general) is a productive form of reflection conducive to dialogue with the world. We used only one subscale modified for our study (7 items) — systemic reflection. Our study applied a 5-point Likert-like scale to evaluate the items.
9. The Satisfaction with Life Scale (SWLS), developed by Diener, Emmons, Larsen and Griffin (1985) and adapted by Leontiev and Osin (2008), has a good reliability level with satisfaction with life. This 5-item scale is assessed according to a 5-point scale depending on the agreement-disagreement with the statement.

It should be noted that the Self-Organisation of Activity Questionnaire and Differential Test of Reflexivity in a modified version (the 5-point Likert-like scale was applied), as described above, as well as the Satisfaction with Life Scale were used to evaluate the study participants’ personal readiness for activity index as an integrative parameter of the psychological system of activity. The personal readiness for activity index is the average of the following indicators: Purposefulness, Planning, Systemic reflection and Life satisfaction, as mentioned above. These transformations were made to develop a research tool adequate to the objective of characterising one’s psychological system of activity via an integrative psychological parameter, namely personal readiness for activity (Atamanova & Bogomaz, 2018; Atamanova et al., 2019).

### Participants

The study was conducted from 2018 to 2019. The initial sample for establishing the cross-cultural variance consisted of 818 people (477 women, 352 men), aged from 18 to 35 (18.32(2.39) M(SD)). Of these, 550 people participated in the study in Russia; 192 in Kazakhstan; 76 in Latvia. The groups differed significantly in age and sex. To compare the indicators, student age and sex were selected from this sample (18– 26 age range).

The final sample (*Table 2*) for comparing the factor structure invariance included 601 people (330 women, 271 men) from Russia (n = 344), Kazakhstan (n = 192), and Latvia (n = 65). The mean age of respondents was 20.33 ± 2.20 years ( Russia = 20.18 years; Kazakhstan = 20.44 years; Latvia = 20.85 years) and 54.9% were women ( Russia = 57.6%; Kazakhstan = 49.5% ; Latvia = 53.8%). The samples did not differ significantly in age (χ^2^(2) = 7.01, p = 0.06) and sex (χ^2^ = 2.866, df = 2, p = 0.239).

All the respondents have lived in their respective country for more than five years. The average period of living in their countries was 17.6±4.8 years (Russia = 17.7 years; Kazakhstan = 16.7 years; Latvia = 19.4 years). In this study, the birth nation does not exclude the respondents, as they might have lived there only for a short period or perhaps were born when their parents stayed in that particular nation and never lived there. The study participants were majoring in various subjects, but this aspect was not in question in this study.

**Table 2:** Demographic characteristics of the Russian, Kazakhstani and Latvian samples

	Russia (n = 344)	Kazakhstan (n = 192)	Latvia (n = 65)
Age (Mean(SD)	20.18 (1.66)	20.44 (2.96)	20.85 (2.01)
Sex (M/F)	146/198	96/96	30/35
Period of living in the country	6–24 years	5–23 years	5–26 years

### Procedure

The present study was designed based on a snowball sampling technique. The study participants were asked to complete a series of questionnaires, their participation in the study was voluntary. The paper-and-pencil forms were offered in Russian. The university students from Kazakhstan and Latvia who participated in the study could understand the Russian language.

### Analysis

The data collected was then statistically treated applying the IBM SPSS Statistics 23. Descriptive analysis and analysis of variance (ANOVA, the F-test criterion), Pearson’s correlation coefficient and stepwise linear regression were used to compare the subsamples. Correlation analysis was performed to determine a significant relationship between value orientations and activity dimensions. In addition, analysis of variance was done to check if there are any significant differences between the three countries among value orientations and activity dimensions. Finally, regression analysis was performed for the personal readiness for activity index in the countries under study, and the six dependent variables: Traditional values versus Secular-rational values, Need for achievement, Need for affiliation, the SEBVR index, the Q-LES-Q index, Subjective happiness, as well as the respondents’ period of living in their countries. Regression analysis was carried out separately for each subsample under the study. The idea was that psychological variables involved in the study could fully characterise young people’s psychological system of activity, when viewed as predictors of their personal readiness for activity index.

## Results

We conducted descriptive statistics (mean and standard deviation) and correlation analysis between value orientations and Personal readiness for activity index for the total sample (n = 601). The results show that out of 13 value orientations, 9 are significantly related to Personal readiness for activity index (see *Table 3*). In general, our findings focus on the relationship between Personal readiness for activity index and Uncertainty avoidance index, Long-term orientation index, Indulgence versus restraint index, Traditional values versus Secular-rational values, Need for achievement, Need for affiliation, SEBVR index, Q-LES-Q index, and Subjective happiness. Power distance index, Individualism index, and Masculinity index were not found to be related to Personal readiness for activity index. However, this relationship could be different in countries yet to be analysed, and more detailed results could be seen in regression analysis.

**Table 3:** Means (M), Standard Deviations (SD), and Correlations

Values	M(SD)	1	2	3	4	5	6	7	9	10	11	12	13
PDI	50.0(18.18)												
IDV	52.2(13.62)	–.067											
MAS	42.4(16.04)	.181**	–.164**										
UAI	47.4(16.91)	.157**	.014	.071									
LTO	57.9(15.64)	.120*	–.217**	.072	.063								
IVR	60.2(13.09)	–.111*	.184**	–.126*	–.213**	–.098							
T/S-RV	4.3(0.98)	.089	–.215**	–.035	–.035	.307**	–.017						
Need for achievement	4(0.67)	.031	–.019	.020	–.054	.131*	.068	.210**					
Need for affliation	4(0.81)	.049	–.022	–.042	–.042	.058	.069	.295**	.431**				
SEBVR index	5.5(1.05)	.054	–.106*	–.021	–.016	.192**	–.009	.238**	.220**	.207**			
Q-LES-Q index	15.6(3.30)	–.024	.009	.017	–.206**	.062	.080	.207**	.231**	.303**	.314**		
Subjective happiness	10.8(2.71)	–.036	–.078	–.087	–.281**	.076	.164**	.331**	.231**	.241**	.281**	.439**	
Personal readiness for activity index	3.6(0.53)	–.002	–.006	–.098	–.116*	.126*	.161**	.371**	.382**	.403**	.204**	.287**	.404**

Note. PDI = Power distance index. IDV = Individualism index. MAS = Masculinity index. UAI = Uncertainty avoidance index. LTO = Long-term orientation index. IVR = Indulgence versus restraint index. T/S-RV = Traditional values versus Secular-rational values. * — p < .01; ** — p < .001.

*Table 4* shows the means, standard deviations, and range for all the variables used in the analysis, grouped across Russia, Kazakhstan, and Latvia. For example, in cultural dimensions, the Individualism index score of Russia is significantly higher than that of Kazakhstan (p = 0.007). While considering the Uncertainty avoidance score of Kazakhstan, it is found to be significantly lower than that of Latvia (p = 0.004). Moreover, the contrast pattern can be seen in Russia (p = 0.00001) and Latvia (p = 0.001), and both have a lower Long-term orientation index than Kazakhstan.

**Table 4:** Analysis of means (M), standard deviations (SD) and variance across countries

Values	Russia M(SD)	Kazakhstan M(SD)	Latvia M(SD)	ANOVA				
				Main effect F; *p* value		Bonferroni post hoc		
						Russia VS Kazakhstan	Russia VS Latvia	Kazakhstan VS Latvia
PDI	50.41(18.12)	48.67(18.42)	52.03(17.83)	.855	.426	.834	1.000	.813
IDV	53.57(13.7)	49.74(13.16)	51.98(13.75)	4.674	**.010**	**.007**	1.000	.711
MAS	43.37(17.07)	41.71(14.51)	44.20(14.72)	.907	.404	.632	1.000	1.000
UAI	47.92(17.17)	44.77(15.51)	52.00(18.41)	5.648	**.004**	.096	.138	**.004**
LTO	56.14(15.84)	62.22(13.75)	54.06(17.23)	11.586	**.000**	**0.000**	1.000	**.001**
IVR	60.50(13.73	59.35(12.08)	60.92(12.55)	.479	.620	1.000	1.000	1.000
T/S-RV	4.07(0.86)	4.72(1.03)	4.02(0.83)	40.473	**.000**	**0.000**	1.000	**0.000**
Need for achievement	4.00(0.66)	4.14(0.64)	3.88(0.63)	6.452	**.002**	**.011**	.524	**.006**
Need for affiliation	4.02(0.82)	4.10(0.76)	3.74(0.89)	4.863	**.008**	1.000	**.018**	**.007**
SEBVR index	5.29(1.15)	5.88(0.79)	5.87(0.79)	23.050	**.000**	**.000**	**.004**	1.000
MPrS	5.47(1.36)	5.95(1.00)	5.97(1.34)	14.414	**.000**	**.000**	**.000**	.761
MPuS	4.64(1.26)	5.28(1.08)	5.05(1.34)	20.812	**.000**	**.000**	**.002**	1.000
MVS	5.77(1.28)	6.21(1.06)	6.09(1.53)	10.402	**.000**	**.000**	**.008**	1.000
MES	5.35(1.36)	5.60(0.79)	5.71(1.42)	19.291	**.000**	**.000**	**.006**	1.000
Q-LES-Q index	15.19(4.0)	16.43(2.24)	15.43(2.35)	18.601	**.000**	**.000**	**.020**	**.000**
Physical health	13.18(3.62)	14.70(2.84)	13.25(3.81)	17.520	**.000**	**.000**	1.000	**.000**
Subjective feelings	19.24(3.76)	20.47(3.56)	19.03(4.05)	11.872	**.000**	**.000**	1.000	**.002**
Leisure time activities	10.66(2.40)	11.13(2.03)	10.60(2.25)	4.542	**.011**	**.014**	1.000	.115
Social relationships	19.13(3.76)	19.40(3.40)	18.86(3.37)	1.177	.309	.651	1.000	.554
Subjective happiness	10.40(2.67)	11.67(2.63)	9.98(2.51)	17.897	**.000**	**.000**	.484	**.000**
Purposefulness	3.91(0.72)	4.11(0.64)	4.01(0.61)	.55	.577	**0.012**	0.882	1.000
Planning	3.31(0.96)	3.37(0.87)	3.27(0.92)	.37	.688	1.000	1.000	1.000
Systemic reflection	3.95(0.60)	3.89(0.56)	3.89(0.48)	.55	.577	0.986	1.000	1.000
Satisfaction with life	3.27(0.74)	3.48(0.79)	2.97(0.80)	11.39	**.000**	**0.016**	**0.018**	**0.000**
Personal readiness for activity index	3.63(0.53)	3.71(0.53)	3.53(0.48)	3.039	**.049**	.386	.386	.052

*Note. PDI = Power distance index. IDV = Individualism index. MAS = Masculinity index. UAI = Uncertainty avoidance index. LTO = Long-term orientation index. IVR = Indulgence versus restraint index. T/S-RV = Traditional values versus Secular-rational values. SEBVR = Subjective Evaluation of Basic Values Realisability. MPrS = Metavalue of professional self-realisation. MPuS = Metavalue of public self-realisation. MVS = Metavalue of vital self-realisation. MES = Metavalue of existential self-realisation. Q-LES-Q = Quality of Life Enjoyment and Satisfaction Questionnaire.*

The Traditional values versus Secular-rational values indicator is significantly higher in the case of Kazakhstan compared to Latvia (p = 0.000001) and Russia (p = 0.000000000001). Kazakhstan also has a higher Need for achievement dimension than both Russia (p = 0.011) and Latvia (p = 0.006). However, Latvian youth had a significantly lower Need for affiliation dimension than youth in Kazakhstan (p = 0.007) and Russia (p = 0.018). All basic metavalues, such as Metavalue of professional selfrealisation, Metavalue of public self-realisation, Metavalue of vital self-realisation and Metavalue of existential self-realisation, are significantly lower in Russia than in Latvia (p = 0.004 for the SEBVR index) and Kazakhstan (p = 0.00004 for the SEBVR index). The quality of life enjoyment and satisfaction index in Russia is significantly lower than in Kazakhstan (p = 0.00006) and Latvia (p = 0.02) and significantly higher in Kazakhstan than in both Russia and Latvia (p = 0.0001).

The parameters of the psychological system of activity show that Purposefulness is significantly lower in Russia than in Kazakhstan (p = 0.012). Satisfaction with life in Latvia is significantly lower than in Kazakhstan (p = 0.00001) and Russia (p = 0.018) and significantly higher in Kazakhstan than in both Russia and Latvia (p = 0.016). The score of the Personal readiness for activity index is higher for youth from Kazakhstan compared to Latvia on a tendency level (p = 0.052).

Stepwise linear regression was conducted to identify which value orientations could be significant predictors of Personal readiness for activity index in each country. The Russian sample collinearity tests confirmed the independence of predictor variables (the tolerance range = 0.80 to 0.96, the VIF range = 1.0 to 1.2), and the assumption of independent errors of residuals was met (the Durbin-Watson value was 1.94) in all four models. The Kazakhstani sample collinearity tests confirmed the independence of predictor variables (the tolerance range = 0.78 to 0.93, the VIF range = 1.28 to 1.1), and the assumption of independent errors of residuals was met (the Durbin-Watson value was 2.08) in all four models. The Latvian sample collinearity tests confirmed the independence of predictor variables (the tolerance range = 0.90 to 0.95, the VIF range = 1.05 to 1.5), and the assumption of independent errors of residuals was met (the Durbin-Watson value was 2.39) in all two models.

*Table 5* demonstrates the overall significance of value orientations and attitudes in Personal readiness for activity across cultures. The need for affiliation is just one significant predictor of Personal readiness for activity index for all samples. The Russian sample regression model (F(1, 315) = 53.05, p < 0.000, R2 = 0.391, R2 Adjusted = 0.383) showed that Need for achievement, Subjective happiness, Traditional values versus Secular-rational values and Need for affiliation were significant predictors of Personal readiness for activity index, accounting for 38% of the variance. Similarly, the Kazakhstani sample regression model also showed that Traditional values versus Secular-rational values (T/S-RV), Need for afiliation and, in addition, the Quality of Life Enjoyment and Satisfaction index (Q-LES-Q index) accounted for 29% of the variance in mean scores for Personal readiness for activity index (F(1, 184) = 25,70, p < 0.000, R2 = 0.294, R2 Adjusted = 0.285). The Latvian sample regression model showed that the Quality of Life Enjoyment and Satisfaction index (Q-LES-Q index), Need for affiliation accounted for 57% of variance in the indicators concerned (F(1, 60) = 41,32, p < 0.000, R2 = 0.579, R2 Adjusted = 0.565).

**Table 5:** Cross-cultural stepwise multiple-regression analyses between the Personal readiness for activity index as a dependent variable and values and attitudes

Model	Predictor variable	b	SE	Beta	p	Adjusted R2 (p)
*Russian sample*						
Model I	Need for achievement	.371	.039	.465	.000	.214 (<.000)
	Constant	2.144	.157		.000	
Model II	Need for achievement	.307	.036	.385	.000	.344 (<.000)
	Subjective happiness	.075	.009	.371	.000	
	Constant	1.615	.157		.000	
Model III	Need for achievement	.299	.035	.374	.000	.379 (<.000)
	Subjective happiness	.061	.010	.302	.000	
	T/S-RV	.118	.029	.190	.000	
	Constant	1.315	.170			
Model IV	Need for achievement	.267	.037	.334	.000	.391(<.011)
	Subjective happiness	.060	.010	.294	.000	
	T/S-RV	.101	.029	.163	.001	
	Need for affiliation	.080	.031	.123	.011	
	Constant	1.209	.174		.000	
*Kazakhstani sample*						
Model I	Need for affiliation	.293	.047	.411	.000	.165 (<.000)
	Constant	2.508	.197		.000	
Model II	Need for affiliation	.239	.047	.335	.000	.233 (<.000)
	T/S-RV	.144	.034	.278	.000	
	Constant	2.041	.219		.000	
Model III	Need for affiliation	.167	.050	.234	.001	.285 (.000)
	T/S-RV	.130	.033	.252	.000	
	Q-LES-Q index	.231	.062	.256	.000	
	Constant	1.542	.250		.000	
*Latvian sample*						
Model I	Q-LES-Q index	.602	.093	.638	.000	.398(<.000)
	Constant	1.490	.317		.000	
Model II	Q-LES-Q index	.514	.081	.546	.000	.565(<.000)
	Need for affiliation	.223	.045	.425	.004	
	Constant	.953	.290		.000	

*Note. T/S-RV = Traditional values versus Secular-rational values. Q-LES-Q = Quality of Life Enjoyment and Satisfaction Questionnaire.*

## Discussion

Cross-cultural comparison of university student samples showed that most differences in the countries in question were between Kazakhstan vs Latvia and Russia in both value orientations and attitudes and Personal readiness for activity index. The youth from Kazakhstan differs significantly in the Long-term orientation index (the highest score) and Uncertainty avoidance index (the lowest score) compared to the youth from Russia and Latvia. In other words, Kazakhstan youth follow strict behavioural codes, rules, and laws, whereas Russian and Latvian youth face fewer regulations and reveal more acceptance of different opinions. Kazakhstan youth, to a larger extent, have the present- and past-looking attribute, whereas Russian and Latvian youth demonstrate more the forward-looking attribute. Our data suggests a long-term orientation of Kazakhstan youth, which is consistent with the results of Minkov et al. (2018). Our study found significant differences in the Individualism versus collectivism dimensions between Kazakhstan and Russian youth. Russian youth have looser ties and emphasise self-interest and ‘I’ consciousness, whereas Kazakhstan youth, to a greater extent, have strong social bonding and emphasise group harmony and ‘We’ consciousness. The results obtained were compared with the ones previously argued that Kazakhstan shared many attributes common to collective cultures (Karibayeva & Kunanbayeva, 2016, Latova, 2016). This demonstrates the differences between collectivistic societies, where cognitive consistency is less important and conforming to situational pressures is more important than in individualistic societies (Suh, 2002; Triandis, 2001). Our conclusion contradicts Temirbekova, Latovb, Latovac & Temirbekovd (2014) who studied 626 students from Russia, Kyrgyzstan, Kazakhstan and Ukraine. The authors revealed that Russian students had the highest power distance, and Kazakhstan students stand out as more individualistic than other countries. Differences can be associated with both the characteristics of the sample and the timing of the study. Russian-speaking young people majoring in different fields took part in our study. In the study from Temirbekova et al. (2014), the study participants majored in business administration. Last but not least, more than five years passed between these two studies, which could affect value orientations.

Kazakhstan youth also differ significantly from Russian and Latvian youth in the Traditional vs Secular-rational values indicator that depicts a continuum where the traditional side is associated with the importance of existential security, traditional family ties, and the strong presence of religion and hierarchy. Residents from Latvia more often choose secular-rational values, and residents of Kazakhstan, traditional values, Russian people occupy an intermediate position between them within the Traditional values versus Secular-rational values continuum.

Furthermore, the differences between Kazakhstan vs Latvia and Russia connected with personal well-being, such as the quality of life enjoyment and satisfaction scales (the total index and the physical health, subjective feelings, and leisure time activities scales), subjective happiness and satisfaction with life. This highlights the importance of collectivist and traditional values and feelings of happiness and well-being among the youth of Kazakhstan.

In addition, higher scores in purposefulness and personal readiness for activity index in youth from Kazakhstan may also connect with their adherence to traditional values compared to those in Russian and Latvian youth. Such value orientations may result in a desire to follow a certain algorithm to achieve one’s purposes.

The study participants from Russia and Latvia have more pronounced individualism dimensions and secular-rational values, but these samples may differ in their motivation. Russian youth seem to be more focused on personal achievements and social support, and they appreciate putting in hard work to achieve goals and the attention of others. Latvian youth showed a higher subjective evaluation of possibilities for realising their basic values in their local settings in the case of Metavalue of professional self-realisation and Metavalue of existential self-realisation. This may signal their greater desire to rely on themselves and a more realistic view of their opportunities.

In general, our data reports on the reasonable links between the Personal readiness for activity index and cultural dimensions, value orientations and psychological indicators, such as Uncertainty avoidance index, Long-term orientation index, Indulgence versus restraint index, Traditional values versus Secular-rational values, Need for achievement, Need for affiliation, SEBVR index, Q-LES-Q index, and Subjective happiness. This could mean that active young people feel happier and more satisfied than passive people. A predisposition to secular-rational values, a desire for affiliation and achievement, a long-term perspective, hardiness and mindfulness characterises them. The mechanisms underlying the relationship between well-being dimensions and personality ones in the context of one’s readiness for activity involve psychological factors. One possible explanation is that happy and successful individuals have more freedom to determine their own life pathways and make choices (Diener, Diener & Diener, 1995). Attribution of success in life to one’s own actions may also contribute to higher levels of well-being (Diener et al., 1995). This is partially consistent with our results; Kazakhstan youth have a more pronounced feeling of happiness and well-being and a higher degree of personal readiness for activity.

We tried to answer the question of what cultural dimensions, value orientations and psychological characteristics could be predictors of personal readiness for activity in Russian, Latvian, and Kazakhstan youth. There was only one dimension: the need for affiliation was common across all countries as a predictor of young people’s personal readiness for activity. However, in all samples, the need for affiliation described a small part of the variance in the mean scores for the Personal readiness for activity index. Personal readiness for activity in Russian students depends on their desire for achievement and level of happiness. In the Kazakhstani and Latvian samples, the most important input was provided by the Quality-of-Life Enjoyment and Satisfaction index. The Russian sample differed more from the samples of Kazakhstan and Latvia, which have more similarities.

## Conclusion

1. Cultural dimensions, traditional versus secular-rational values, need for achievement and affiliation, feelings of happiness and well-being have medium and high correlations with personal readiness for activity.
2. Cross-cultural variance was established for most values and cultural dimensions in the Russian, Kazakhstani, and Latvian samples. The cross-cultural similarity was established in the psychological system of activity for the three countries; personal readiness for activity was different on a tendency level.
3. Need for affiliation is common in all countries as a predictor of young people’s personal readiness for activity, but it describes a small part of the variance. The Russian university students’ personal readiness for activity depends on their need for achievement and level of happiness. In the Kazakhstani and Latvian samples, the most important input was provided by the Quality-of-Life Enjoyment and Satisfaction index.

To sum up the study outcomes, it should be noted that the study focus was on examining the relationships between value orientations, attitudes and the psychological system of activity in young people in post-Soviet countries. To reveal cross-cultural differences, if any, in psychological variables in question and relationships between them, three samples of youth (Russia, Kazakhstan and Latvia) who do not differ in age or gender were involved in the study concerning the period they had lived in the countries. Our research continues the tradition of cross-cultural studies investigating cultural diversity in the post-Soviet space (Minkov et al., 2018; Becker et al., 2018) and expands the research by considering youth as a platform representing social progress, human capital and socio-economic and political development (Lebedeva & Tatarko, 2019; Atamanova et al., 2019). We have found that value orientations affect personal readiness for activity. However, there are more differences in these relationships than similarities between youth from Russia, Kazakhstan and Latvia. It is important to consider the cross-cultural value differences in the context of youth’s personal and professional development.

## Limitations

One of the limitations of this study is that all the participants were Russian-speaking young people. Differences probably exist in value orientations and parameters of the psychological system of activity among Kazakhstan and Latvian youth who do not speak the Russian language. The second limitation concerns the generalisability of the findings because of the study design (a snowball sampling technique was applied). In order to achieve more robust results in cross-cultural research, the selected samples should include participants from a wider range of social and regional groups.

## References

[ref1] Ajzen, I. (1988). Attitudes, Personality, and Behavior. Chicago, IL: Dorsey Press.

[ref2] Alimbayeva, R., Baimukanova, M., Sabirova, R., Karipbaev, B., & Tamabayeva, M. (2018). Psychological peculiarities of the professional self-determination of social orphans in senior adolescence. International Journal of Adolescence and Youth, 23(4), 457–467. 10.1080/02673843.2018.1433694

[ref3] Atamanova, I., Bogomaz, S., & Filippova, T. (2019). Modern youth’s value orientations and activity in a cross-cultural context. 7th International Interdisciplinary Scientific Conference «Society. Health. Welfare» (Riga, Latvia, 10–12 October 2018). SHS Web of Conferences, 68, 01005. 10.1051/shsconf/20196801005

[ref4] Atamanova, I., & Bogomaz, S. (2018). Lichnostnaya gotovnost’ sovremennoi vuzovskoi molodezhi k deyatel’nosti [Modern university youth’s personal readiness for activity]. In Yu.P. Povarenkov (Ed.), Sistemogenez uchebnoi i professional’noi deyatel’nosti. Chast’ I: materialy VIII vserossiyskoi nauchnoprakticheskoi konferentsii [Systemogenesis of educational and professional activity. Part I: Proceedings of the VIII All-Russian scientific-practical conference] (Yaroslavl, 19–20 November 2018) (pp. 134–137). Yaroslavl: YSPU Editorial and Publishing Department.

[ref5] Azimbayeva, G. (2017). Comparing post-Soviet changes in higher education governance in Kazakhstan, Russia, and Uzbekistan. Cogent Education, 4. 10.1080/2331186X.2017.1399968

[ref6] Baumann, C., & Winzar, H. (2017). Confucianism and work ethic – introducing the ReVAMB model. In I. Oh & G.S. Park (Eds.), The Political Economy of Business Ethics in East Asia: A Historical and Comparative Perspective (pp. 33–60). Amsterdam: Elsevier-Chandos. 10.1016/B978-0-08-100690-0.00003-8

[ref7] Becker, M., Vignoles, V. L., Owe, E., Easterbrook, M. J., Brown, R., Smith, P. B., ... & Lay, S. (2018). Being oneself through time: Bases of self-continuity across 55 cultures. Self and Identity, 17(3), 276–293. 10.1080/15298868.2017.1330222

[ref8] Bell, M.P., Connerley, M.L., & Cocchiara, F.K. (2009). The case for mandatory diversity education. Academy of Management Learning & Education, 8(4), 597–609. 10.5465/AMLE.2009.47785478

[ref9] Bogomaz, S., Kozlova N., & Atamanova I. (2015). University students’ personal and professional development: The socio-cultural environment effect. Procedia – Social and Behavioral Sciences, 214, 552–558. 10.1016/j.sbspro.2015.11.759

[ref10] Bogomaz, S.A. (2014). Innovatsionnyi potentsial lichnosti i ego otsenka [A person’s innovative potential and its assessment]. In N.I. Leonov (Ed.), A man’s social world: Proceedings of V All-Russian scientific-practical conference with foreign researchers’ participation «A man and the world: Psychology of conflict, uncertainty and risk of innovation» 17–19 April 2014 (pp. 275–279). Izhevsk: ERGO.

[ref11] Bogomaz, S.A., Boyko, E.A., & Yashina, V.V. (2019). Vzaimosvyaz emotsionalnogo intellekta s para-metrami deyatelnosti, motivatsii i tsennostey u rossiyskoy vuzovskoy molodezhi [The relationship between Russian university youth’s emotional intelligence and their parameters of activity, motivation and values]. Psikhologiya kognitivnykh protsessov [Psychology of cognitive processes], 8, 14–25. Retrieved from https://www.elibrary.ru/download/elibrary_42374393_56015908.pdf

[ref12] Buravleva, N., & Bogomaz, S. (2020) Readiness for innovative activities among students of technical universities. Russian Psychological Journal, 17(3), 30–43. 10.21702/rpj.2020.3.3

[ref13] Diener, E., Emmons, R., Larsen, R., & Griffin, S. (1985). Intensity and frequency: Dimensions underlying positive and negative affect. Journal of Personality Assessment, 49, 71–75. 10.1037/0022-3514.48.5.1253 3998989

[ref14] Diener, E., Diener, M., & Diener, C. (1995). Factors predicting the subjective well-being of nations. Journal of Personality and Social Psychology, 69, 851–864. 10.1037/0022-3514.69.5.851 7473035

[ref15] Fedotova, V.A. (2017). Age-related differences in values and economic attitudes among Russians. Psychology in Russia: State of the Art, 10(1), 105–116. 10.11621/pir.2017.0108

[ref16] Filenko, I.A., Atamanova, I.V., & Bogomaz, S.A. (2020). Examining the psychometric characteristics of the subjective evaluation of basic values realizability (SEBVR) technique designed for studying value orientations. Bulletin of Kemerovo State University, 22(4), 1028-1039. 10.21603/2078-8975-2020-22-4-1028-1039

[ref17] Glasman, L.R., & Albarracín, D. (2006). Forming attitudes that predict future behavior: A meta-analysis of the attitude-behavior relation. Psychological Bulletin, 132(5), 778–822. 10.1037/0033-2909.132.5.778 16910754 PMC4815429

[ref18] Halstead, J.M., & Taylor, M. J. (2000). Learning and teaching about values: A review of recent research. Cambridge Journal of Education, 30(2), 169–202. 10.1080/713657146

[ref19] Hofstede, G., & Minkov, M. (2013). Values survey module 2013. Retrieved from http://geerthofstede.com/wp-content/uploads/2016/07/Manual-VSM-2013.pdf

[ref20] Hofstede, G. (1983). National cultures revisited. Behavior Science Research, 18(4), 285–305. 10.1177/106939718301800403

[ref21] Homan, A.C., van Knippenberg, D., Van Kleef, G.A., & De Dreu, C.K.W. (2007). Bridging fault-lines by valuing diversity: diversity beliefs, information elaboration, and performance in diverse work groups. Journal of Applied Psychology, 92(5), 1189–1199. 10.1037/0021-9010.92.5.1189 17845079

[ref22] Inglehart, R., & Welzel, C. (2005). Modernization, cultural change, and democracy: The human development sequence. New York: Cambridge University Press.

[ref23] Inglehart, R. (1997). Modernization, postmodernization and changing perceptions of risk. International Review of Sociology, 7(3), 449–459. 10.1080/03906701.1997.9971250

[ref24] Jackson, D.N. (1984). Personality Research Form Manual. Port Huron: Research Psychologists Press.

[ref25] Karibayeva, B., & Kunanbayeva, S.S. (2017). Power distance and verbal index in Kazakh business discourse. International Journal of Speech Technology, 20(4), 779–785. 10.1007/s10772-017-9450-0

[ref26] Khabibulin, R.K. (2015). Characteristics of the Russian state power authority in the citizens’ minds: Ph. D. dissertation (Psychology). Saint Petersburg, Retrieved from https://rusneb.ru/catalog/000199_000009_005569000/

[ref27] Khabibulin, I.M. (1999). Vremennaya perspektiva i motivatsionnyie predpochteniya [Time perspective and motivational preferences]. Journal of Applied Psychology, 4, 99–113.

[ref28] Kwon, S.W., Heflin, C., & Ruef, M. (2013). Community social capital and entrepreneurial activity. American Sociological Review, 78(6), 980–1008. 10.1177/0003122413506440

[ref29] Latova, N. (2016) Kulturnaya spetsifika rossiyan (etnometricheskiy analiz na osnove kontseptsii G.Hofsteda) [The industrial culture of modern Russian workers as an element of their human capital: an ethnometric analysis using Hofstede’s model]. Mir Rossii, 26(3), 36–63.

[ref30] Lauring, J., & Selmer, J. (2013). Diversity attitudes and group knowledge processing in multicultural organizations. European Management Journal, 31(2), 124–136. 10.1016/j.emj.2012.03.016

[ref31] Lebedeva, N.M, & Tatarko, A.N. (2019). Vektoryi razvitiya stran v edinom prostranstve kulturnyih izmereniy [The vectors of development of the countries in a combined space cultural dimensions]. Psikhologicheskii zhurnal, 40(6), 99–111. 10.31857/S020595920007368-0

[ref32] Leontiev, D.A., & Osin, E.N. (2014). Refleksiya «horoshaya» i «durnaya»: ot ob’yasnitelnoy modeli k differentsialnoy diagnostike [“Good” and “bad” reflection: From an explanatory model to differential assessment]. Psychology. Journal of the Higher School of Economics, 11(4), 110–135. Retrieved from https://psy-journal.hse.ru/2014-11-4/141399859.html

[ref33] Leontiev, D.A. (2011). Lichnostnyi potentsial: Struktura i diagnostika [Personal potential: Structure and diagnosis]. Moscow: Smysl.

[ref34] Leyden D.P., & Link A.N. (2015). Toward a theory of the entrepreneurial process. Small Business Economics, 44(3), 475–484. 10.1007/s11187-014-9606-0

[ref35] Lyubomirsky, S., & Lepper, H.S. (1999). A measure of subjective happiness: Preliminary reliability and construct validation. Social Indicators Research, 46(2), 137–155. 10.1023/A:1006824100041

[ref36] Mandrikova, E.Yu. (2010). Oprosnik samoorganisatsii deyatel’nosti [The self-organization of activity questionnaire]. Psychological Diagnostics, 2, 87–111. Retrieved from https://publications.hse.ru/mirror/pubs/share/folder/i41i2cox7a/direct/73401318.pdf

[ref37] Minkov, M., Bond, M. H., Dutt, P., Schachner, M., Morales, O., Sanchez, C., Jandosova J., Khassenbekov Y., & Mudd, B. (2018). A reconsideration of Hofstede’s fifth dimension: New flexibility versus monumentalism data from 54 countries. Cross-Cultural Research, 52(3), 309–333. 10.1177/1069397117727488

[ref38] Mor Barak, M.E. (2017). Managing diversity: Toward a globally inclusive workplace (4th ed.). Thousand Oaks, CA: Sage CA.

[ref39] Mykhailenko, O., Blayone, T.J.B., Usca, S., Kvasovskyi, O., & Desyatnyuk, O. (2020). Optimism, interest and gender equality: comparing attitudes of university students in Latvia and Ukraine toward IT learning and work. Compare: A Journal of Comparative and International Education. 10.1080/03057925.2020.1843999

[ref40] Osin, E.N., & Leontiev, D.A. (2020) Kratkie russkoyazyichnyie shkalyi diagnostiki sub’ektivnogo blagopoluchiya: psihometricheskie harakteristiki i sravnitelnyiy analiz [Brief Russian-language instruments to measure subjective well-being: psychometric properties and comparative analysis] Monitoring of public opinion: economic and social changes journal, 1, 117–142. 10.14515/monitoring.2020.1.06

[ref41] Osin, E. N., & Leontiev, D. A. (2008). Aprobatsiya russkoyazychnykh versii dvukh shkal ekspressotsenki sub’ektivnogo blagopoluchiya [Aprobation of Russian versions of express-evaluation scales of subjective well-being]. Materialy III Vserossiiskogo Sotsiologicheskogo Kongressa. Moscow: Institute of Sociology of the Russian Academy of Sciences / Russian Society of Sociologists. Retrieved from http://www.isras.ru/abstract_bank/1210190841.pdf

[ref42] Porter, M.E., Stern, S., & Green, M. (2017). Social progress index 2017. Social Progress Imperative. Retrieved from https://www2.deloitte.com/content/dam/Deloitte/co/Documents/about-deloitte/Social-Progress-Index-2017.pdf

[ref43] Rasskazova E. (2012). Metodika otsenki kachestva zhizni i udovletvorennosti: psihometricheskie harakteristiki russkoyazyichnoy versii [Evaluation of quality of life enjoyment and satisfaction: Psychometric properties of a Russian-language measure]. Psychology. Journal of the Higher School of Economics, 9(4). 81–90. Retrieved from https://psy-journal.hse.ru/en/2012-9-4/68135939.html

[ref44] Ritsner, M., Kurs, R., Gibel, A., Ratner, Y., & Endicott, J. (2005). Validity of an abbreviated quality of life enjoyment and satisfaction questionnaire (Q-LES-Q-18) for schizophrenia, schizoaffective, and mood disorder patients. Quality of Life Research, 14(7), 1693–1703. 10.1007/s11136-005-2816-9 16119181

[ref45] Schwartz, S.H. (1994). Beyond individualism/collectivism: New cultural dimensions of values. In U. Kim, H. C. Triandis, Ç. Kâgitçibasi, S.-C. Choi, & G. Yoon (Eds.), Cross-cultural research and methodology series, Vol. 18. Individualism and collectivism: Theory, method, and applications (pp. 85–119). Sage Publications, Inc.

[ref46] Suh, E.M. (2002). Culture, identity consistency, and subjective well-being. Journal of Personality and Social Psychology, 83, 1378–1391. 10.1037/0022-3514.83.6.1378 12500819

[ref47] Taber, K.S. (2018). The use of Cronbach’s alpha when developing and reporting research instruments in science education. Research in Science Education, 48(6), 1273–1296. 10.1007/s11165-016-9602-2

[ref48] Temirbekova, Z., Latov, Y.V., Latova, N.V., & Temirbekov, Z. (2014). Work related values: a comparison of four post-soviet countries. Procedia – *Social and Behavioral Sciences,* 109, 794–798. 10.1016/j.sbspro.2013.12.545

[ref49] Triandis, H.C. (2001). Individualism-collectivism and personality. Journal of Personality, 69, 907–924. 10.1111/1467-6494.696169 11767823

[ref50] Yakavets, N. (2016). Societal culture and the changing role of school principals in the post-Soviet era: The case of Kazakhstan. Journal of Educational Administration, 54(6), 683–702. 10.1108/JEA-12-2015-0118

